# Prognostic factors in oropharyngeal squamous cell carcinoma in the state of São Paulo, Brazil: 10-year follow-up

**DOI:** 10.1016/j.bjorl.2022.07.003

**Published:** 2022-08-05

**Authors:** Fabio Lau, Jonas Belchior Tamanini, Fabio Portella Gazmenga, Gustavo Mercuri, Vanessa Carvalho de Oliveira, Daniel Naves Araújo Teixeira, Eduardo Vieira Couto, Carlos Takahiro Chone

**Affiliations:** Universidade Estadual de Campinas (Unicamp), Departamento de Otorrinolaringologia e Cirurgia de Cabeça e Pescoço, São Paulo, SP, Brazil

**Keywords:** Squamous cell carcinoma, Oropharynx, Survival

## Abstract

**Objective:**

To descriptively analyze the epidemiological data, clinical stage, and outcomes of oropharyngeal squamous cell carcinoma in the state of São Paulo, Brazil, and to estimate the influence of clinical stage and treatment type on overall and disease-free survival.

**Methods:**

We retrospectively analyzed epidemiological data from the São Paulo Cancer Center Foundation database relative to patients with oropharyngeal squamous cell carcinoma diagnosed between 2004 and 2014 in the state of São Paulo. Univariate and multivariate Cox regression analyses were performed to assess factors associated with the outcomes. A forward stepwise selection procedure was used. Survival curves were estimated by the Kaplan-Meier method and compared by the Gehan-Breslow-Wilcoxon test.

**Results:**

A total of 8075 individuals with oropharyngeal squamous cell carcinoma were identified. Of these, 86.3% were diagnosed at an advanced stage and 13.7% at an early stage. Only 27.2% of patients were treated surgically, whereas 57.5% were treated medically. Patients undergoing surgery had longer overall survival than those receiving medical treatment in both early- and advanced-stage oropharyngeal squamous cell carcinoma. However, there was no significant difference in disease-free survival between surgical and medical treatment.

**Conclusion:**

No significant difference in disease-free survival between medical and surgical treatment suggests similar complete remission rates with both approaches. Patients receiving medical treatment had shorter overall survival, which may be due to complications from chemotherapy and radiotherapy. However, we cannot confirm this relationship based on the data provided by the São Paulo Cancer Center Foundation. Prospective studies are warranted to assess whether the lower overall survival rate in patients receiving medical treatment is secondary to complications from chemotherapy and radiotherapy.

**Level of evidence:**

2C.

## Highlights

• Among patients with oropharyngeal squamous cell carcinoma, 86.3% had advanced-stage disease and 13.7% had early-stage disease at diagnosis.
• Disease-free survival did not differ significantly between patients receiving medical and surgical treatment among patients with the same staging.
• Patients receiving medical treatment had shorter overall survival in both early and advanced stages of the disease compared with those undergoing surgery.

## Introduction

Head and neck neoplasms are the sixth most common type of cancer worldwide with approximately 630,000 new cases annually, resulting in more than 350,000 deaths every year. Considering geographical variations due to sociocultural differences, approximately 10% of these neoplasms are Oropharyngeal Squamous Cell Carcinoma (OPSCC).^1^, ^2^ Squamous Cell Carcinoma (SSC) accounts for more than 90% of all neoplasms of the oral cavity and pharynx.^3^ OPSCC is more common in men than in women,^3^, ^4^, ^5^, ^6^ with a peak incidence in the sixth and seventh decades of life.^6^

The development of OPSCC is associated with factors both intrinsic and extrinsic to the individual. Intrinsic factors include age, race, sex, and genetic susceptibility, whereas extrinsic factors include alcohol consumption, tobacco use, Human Papillomavirus (HPV) infection, diet low in fruit and vegetables, consumption of mate tea, and poor oral hygiene.^6^, ^7^, ^8^, ^9^, ^10^, ^11^, ^12^, ^13^, ^14^, ^15^, ^16^ Historically, the most significant risk factors for OPSCC are smoking and drinking. However, unlike other head and neck neoplasms, the incidence of OPSCC continues to increase, especially in HPV-positive patients.^1^, ^17^, ^18^, ^19^, ^20^, ^21^, ^22^ Patients with HPV-negative OPSCC have an important history of tobacco use and alcohol consumption, unlike patients with HPV-positive OPSCC who tend to be younger, male, white, and from high-income countries.^4^, ^23^, ^24^ Patients with HPV-positive OPSCC also have a better prognosis and survival than those with HPV- negative OPSCC, regardless of the type of treatment.^25^, ^26^ The striking difference in prognosis for HPV-positive vs HPV-negative OPSCC led to the creation of new AJCC staging criteria in 2018, which separate virus-associated and non-virus-associated tumors.

The optimal treatment for patients with OPSCC remains controversial. Currently, there are several treatment options, each with different limitations, sequelae, and outcomes.^7^, ^27^, ^28^, ^29^, ^30^, ^31^, ^32^ Treatment selection is individualized based on tumor stage, anatomic site, recently published articles, center experience, patient preferences, and general characteristics, such as patient age, occupation, performance status, and comorbidities.^7^, ^32^, ^33^, ^34^, ^35^ Patients with early-stage OPSCC have similar outcomes to Radiotherapy (RT) or surgery.^29^, ^33^, ^36^, ^37^, ^38^, ^39^ However, most patients have advanced-stage disease at diagnosis,^40^, ^41^ and there is no consensus on the best management of these cases. Different cancer centers use surgery, RT, and Chemotherapy (CT) in a variety of combinations.^7^, ^27^, ^29^, ^30^^,^^32^

Many issues remain unresolved concerning OPSCC, especially regarding the optimal treatment strategy. Therefore, the aim of the present study was to analyze epidemiological data from patients with OPSCC and compare the outcomes of surgical and medical treatment according to OPSCC stage at diagnosis in the cancer network of the state of São Paulo, Brazil.

## Methods

We retrospectively analyzed epidemiological data obtained from the São Paulo Cancer Center Foundation (*Fundação Oncocentro de São Paulo*, or FOSP for short, in Portuguese) database relative to patients with OPSCC diagnosed between 2004 and 2014 in the state of São Paulo. The following variables were analyzed: sex; age; clinical stage, divided into early (stage I or II) and advanced (stage III or IV); and type of treatment, divided into surgical (surgery alone, surgery and RT, surgery and CT, surgery with RT and CT, surgery with RT, CT, and hormone therapy), medical (RT alone, CT alone, RT and CT), other combinations, and no treatment performed.

The database is available on the FOSP official website at http://www.fosp.saude.sp.gov.br/publicacoes/rhc. The data are in the public domain and not nominal. In accordance with the policy of the Brazilian National Research Ethics Committee (CEP/PRP No. 068/202), studies using publicly available datasets are exempt from institutional research ethics committee approval as they do not involve human subjects.

For inclusion of patients, we selected the anatomic sites for oropharyngeal cancer development based on topographic diagnosis according to the International Classification of Diseases for Oncology (ICD-O) second edition until the end of 2005, and ICD-O third edition from 2006 onward. Initially, all patients listed in the FOSP database with an ICD-O code corresponding to a neoplasm in an anatomic site related to the oropharynx were eligible for inclusion. Subsequently, patients without a diagnosis of OPSCC were excluded.

For descriptive statistics, categorical variables were expressed as numbers (n) and percentages (%), and numerical variables as mean (SD) or median (minimum and maximum values). Univariate and multivariate Cox regression analyses were performed to assess factors associated with the outcomes. A forward stepwise selection procedure was used. Survival curves were estimated by the Kaplan-Meier method and compared by the Gehan-Breslow-Wilcoxon test. The level of significance was set at 5% for all analyses.

## Results

**Figure 1:**
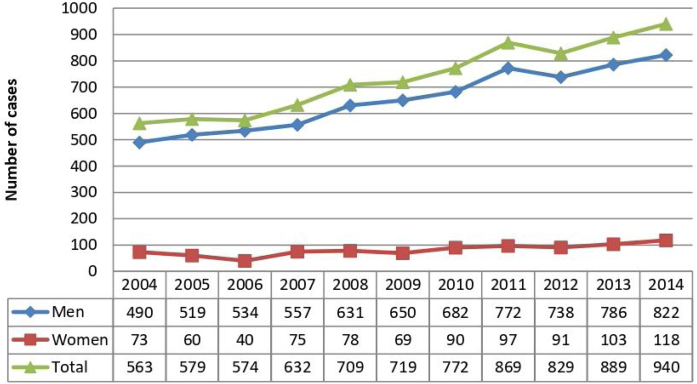
Incidence of oropharyngeal squamous cell carcinoma in the state of São Paulo, Brazil, 2004‒2014.

A total of 8075 patients with OPSCC were identified in the state of São Paulo, with an increasing incidence from 2004 to 2014; 7181 were men (88.9%) and 894 were women (11.1%) (Fig. 1). The mean patient age was 57.96 (SD, ±10.14) years. Patients aged 50–59 years were the most affected (n = 3181, 39.4%), followed by patients aged 60–69 years (n = 2210, 27.4%) and those aged 40–49 years (n = 1424, 17.6%). Therefore, patients aged 40–69 years accounted for 84.4% of cases, whereas those below 40 years of age accounted for only 2.2% of cases.

Patients were divided into 2 groups according to OPSCC stage at diagnosis: early (stage I or II) and advanced (stage III or IV). However, there was no record of this information for 290/8075 patients. Among the remaining 7785 patients, 86.3% were diagnosed at an advanced stage and 13.7% at an early stage. The mean time from diagnosis to initiation of treatment was 69.21 days. This indicates a delay in treatment initiation even after a diagnosis has been reached.

Data on OPSCC type of treatment and reason for untreated patients are shown in Table 1. Only 27.2% of patients were treated surgically, whereas 57.5% were treated medically. In 416 cases (5.2%), patients received other treatment combinations, but it was not recorded whether or not surgery was performed.

**Table 1:** Number of cases of oropharyngeal squamous cell carcinoma according to type of treatment and reasons for untreated patients in the state of São Paulo, Brazil, 2004‒2014.

Treatment		
Type	Nº cases	Freq. (%)
Surgical	2200	27.2
Non-surgical	4640	57.5
None	819	10.1
Other combinations	416	5.2

Untreated patients		
Reason	Nº cases	Freq. (%)
Patient refusal	12	0.1
Advanced disease/Lack of clinical conditions	112	1.4
Comorbities	11	0.1
Treatment abandonment	17	0.2
Death from cancer	448	5.5
Death from another causes	35	0.4
Others	147	1.8
No information	44	0.5
Treated	7249	89.8

No treatment was performed to 819 patients (10.1%). In 483 cases (5.9%), patient died before treatment, 448 (5.5%) from cancer and 35 (0.4%) for other causes; 112 (1.4%) did not undergo treatment due to advanced disease or lack of clinical conditions; 11 (0.1%) due to other comorbities; 17 (0.2%) dropped out and 12 (0.1%) refused treatment. There was no information in 44 (0.5%) cases; and 147 (1.8%) were classified as other causes.

The results of univariate and multivariate Cox regression analyses performed to identify factors related to death and overall recurrence are shown in Table 2, Table 3, respectively.

**Table 2:** Results of Cox regression analysis to estimate the factors associated with death in patients with oropharyngeal squamous cell carcinoma in the state of São Paulo, Brazil, 2004‒2014.

Variable	Category	Univariate analysis			Multivariate analysis		
		*p*-value	HR	95% CI	*p*-value	HR	95%CI
Sex	M × F	<0.0001	1.296	1.192‒1.410	<0.0001	1.253	1.145‒1.373
Stage	Advanced × Early	<0.0001	2.062	1.896‒2.241	<0.0001	1.822	1.666‒1.993
Type of treatment	Medical × Surgical	<0.0001	1.737	1.634‒1.847	<0.0001	1.614	1.514‒1.721
Overall recurrence	Yes × No	<0.0001	1.298	1.217‒1.384	<0.0001	1.132	1.060‒1.210
Age	Numerical range	<0.0001	1.296	1.192‒1.410	<0.0001	1.253	1.145‒1.373
Diagnosis time	Numerical range	<0.0001	1.000	0.999‒1.000	<0.0001	0.998	0.998‒0.999

HR, Hazard Ratio for death; 95% CI, Confidence Interval for the ratio; Diagnosis time, Time from diagnosis to initiation of treatment.

**Table 3:** Results of Cox regression analysis to estimate the factors associated with overall recurrence in patients with oropharyngeal squamous cell carcinoma in the state of São Paulo, Brazil, 2004‒2014.

Variable	Category	Univariate analysis			Multivariate analysis		
		*p*-value	HR	95% CI	*p*-value	HR	95%CI
Sex	M × F	0.6319	1.041	0.884‒1.225			
Stage	Advanced × Early	<0.0001	1.522	1.307‒1.773	<0.0001	1.513	1.293‒1.770
Type of treatment	Medical × Surgical	0.5865	1.031	0.923‒1.152	0.4166	0.954	0.851‒1.069
Age	Numerical range	0.2718	0.997	0.991‒1.002			
Diagnosis time	Numerical range	0.7821	1.000	0.999‒1.000			

HR, Hazard Ratio for death; 95% CI, Confidence Interval for the ratio; Diagnosis time, time from diagnosis to initiation of treatment.

Univariate analysis showed that age, sex, clinical stage at diagnosis, type of treatment, and overall recurrence were significantly associated with death as an outcome. Clinical stage at diagnosis was the most relevant variable, with a Hazard Ratio (HR) of 2.062. The comparison of types of treatment showed an HR of 1.737 for death among patients receiving medical treatment compared with those receiving surgical treatment. Men and patients with recurrence were also more likely to die, with an HRs of 1.296 and 1.298, respectively. The results of the multivariate analysis supported the findings of the univariate analysis. Advanced-stage OPSCC had an HR of 1.822 in relation to early-stage disease. Patients receiving medical treatment were more likely to die than those undergoing surgery, with an HR of 1.614. Men and patients with recurrence were also more likely to die, with an HRs of 1.253 and 1.132, respectively.

Regarding disease-free survival, univariate analysis showed no statistically significant differences for the variables sex, age, time from diagnosis to initiation of treatment, or type of treatment. However, patients with advanced-stage OPSCC were more likely to have overall recurrence than those with early-stage disease in both univariate and multivariate analyses, with an HRs of 1.522 and 1.513, respectively (*p* < 0.0001).

**Figure 2:**
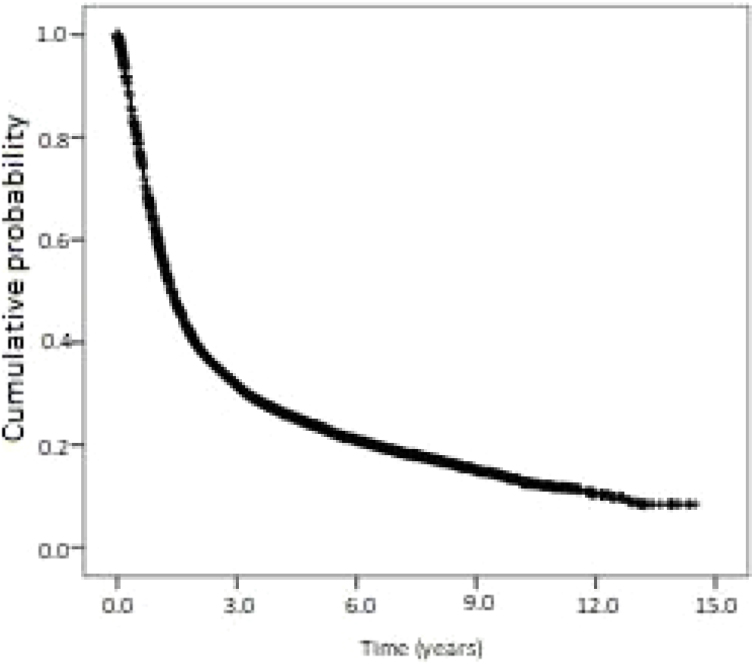
Kaplan-Meier curve of overall survival (all-cause mortality) for patients with oropharyngeal squamous cell carcinoma in the state of Sao Paulo, Brazil, 2004‒2014. Cumulative survival (standard error): 1-year: 60.6% (0.5%); 5-years: 23.7% (0.5%); 10 years: 13.4% (0.6%).

**Figure 3:**
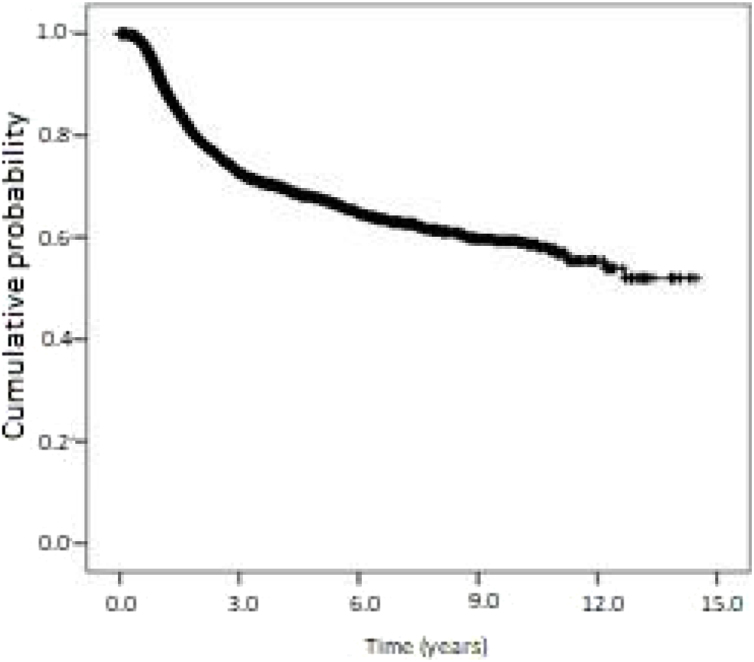
Kaplan-Meier curve of disease-free survival (all-cause mortality) for patients with oropharyngeal squamous cell carcinoma in the state of São Paulo, Brazil, 2004‒2014. Cumulative survival (standard error): 1-year: 91.1% (0.4%); 5-years: 67.7% (0.8%); 10-years: 59.0% (1.3%).

Kaplan-Meier curves of overall survival and disease-free survival are shown in Figure 2, Figure 3. Overall survival was 60.6% at 1-year, 23.7% at 5-years, and 13.4% at 10-years. Disease-free survival was 91.1%, 67.7%, and 59.0% at 1, 5 and 10-years, respectively.

**Figure 4:**
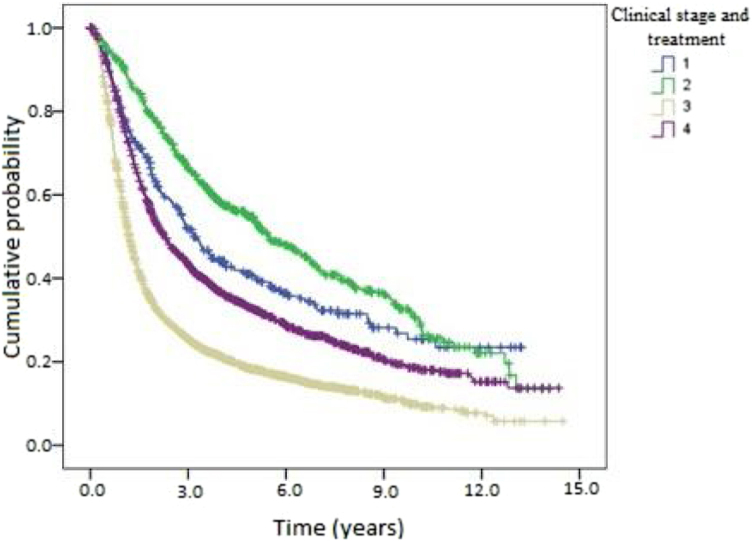
Kaplan-Meier curve of death according to clinical stage and treatment for patients with oropharyngeal squamous cell carcinoma in the state of São Paulo, Brazil, 2004‒2014.

**Figure 5:**
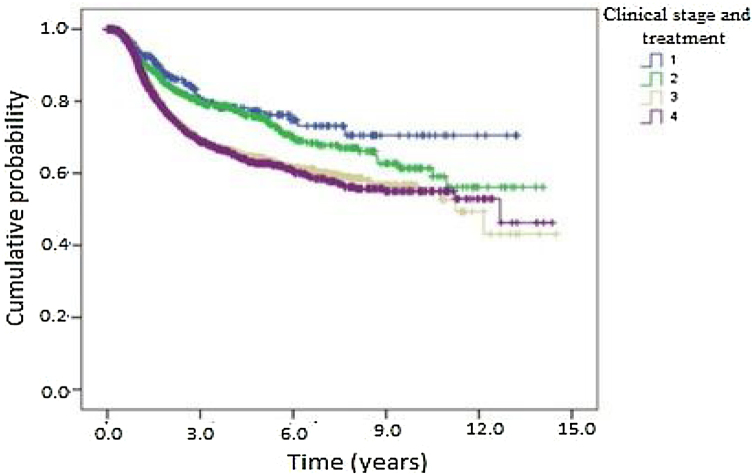
Kaplan-Meier curve of disease-free survival according to clinical stage and treatment for patients with oropharyngeal squamous cell carcinoma in the state of São Paulo, Brazil, 2004‒2014.

Kaplan-Meier curves of death and overall recurrence according to clinical stage and treatment were used to determine the influence of these factors on overall survival and disease-free survival. Patients were stratified into 4 groups according to clinical stage and treatment as follows: early and non-surgical; early and surgical; advanced and non-surgical; and advanced and surgical. There was a significant difference between the groups in overall survival (Fig. 4). Patients with early-stage OPSCC had better results than those with advanced-stage disease, regardless of treatment type. Patients treated medically had shorter overall survival in both early and advanced stages. Fig. 5 shows the results of the comparison of the same 4 groups for disease-free survival. There was a statistically significant difference between clinical stages, where patients with early-stage OPSCC had better results than those with advanced-stage disease, regardless of treatment type. However, there was no significant difference between the groups with the same clinical stage at diagnosis when comparing surgical vs. medical treatment.

## Discussion

The treatment of OPSCC is historically controversial and there is no universally consolidated protocol to guide treatment decision-making.^28^, ^33^, ^42^ Several approaches have been described using different combinations, such as RT alone, surgery alone, and adjuvant or neoadjuvant RT, combined or not with CT, in addition to neck dissection, which can be radical, selective, or even elective.^32^, ^33^, ^41^ The National Head and Neck Cancer Audit recognizes the variation in treatment strategies for OPSCC across cancer networks in England and Wales.^33^ Surgical techniques are also a matter of debate due to the different morbidities that might result from them.^33^, ^43^ The ultimate goal of treatment is cure with minimal functional and aesthetic morbidity.^1^, ^7^

Until the 1990s, open surgery was the primary treatment for OPSCC because of limited access to this complex anatomic site. After the 1990s, concurrent CT and RT gained popularity because of the potential morbidity involved in open surgery.^32^, ^35^, ^44^, ^45^

However, if salvage surgery is required after RT, it will be a more technically challenging procedure due to tissue edema and fibrosis, with an increased risk of postoperative complications and poor wound healing.^46^, ^47^, ^48^ Sassler et al.^48^ found major wound complications in 61% of patients undergoing salvage surgery after completing CT and RT. Complications of Chemoradiotherapy (CRT) include mucositis, fibrosis, xerostomia, dermatitis, osteoradionecrosis, neutropenia, and dysphagia.^49^, ^50^, ^51^, ^52^, ^53^ Studies have also demonstrated severe late toxicity^54^ and high rates of gastrostomy tube dependence in patients treated with CRT.^49^

Primary RT and surgery have been shown to be equally effective in patients with early-stage OPSCC^33^, ^36^, ^38^, ^39^ and are recommended by the U.S. National Comprehensive Cancer Network (NCCN).^36^ In view of similar oncologic outcomes, the complications and functional outcomes associated with each modality gain importance in the choice of treatment. Many centers have opted for organ preservation protocols using RT due to complications secondary to surgery.^31^, ^35^ However, postoperative adverse effects are mainly caused by access to the oropharynx through a transmandibular or transfacial approach.^32^, ^33^, ^50^ Transoral Surgery (TORS) have been developed to reduce surgical morbidity, with 5-year survival rates similar to those of RT in patients with early-stage OPSCC.^38^, ^39^ Althoug some studies suggest superior functional outcomes with TORS compared to CRT or RT, 55, 56 a more recently published randomize trial compared swallow-related outcomes in patients with T1-T2 N0-N2 (LN  ≤ 4 cm) treated with primary RT versus those treated with TORS and showed that RT arm had better outcomes. Nevertheless, this difference was not clinically meaningful and became less pronounced with the passage of time.^57^

Single modality therapy is recommended for patients with early-stage OPSCC to avoid the adverse effects of combined modality therapy.^32^, ^33^, ^36^ In this context, given the potential late adverse effects of RT, TORS is highly promising. Furthermore, surgical resection of the tumor allows for adequate histopathologic staging of the neoplasm and identification of patients who would benefit from adjuvant therapy.^32^, ^33^ If pathologic analysis of the resected tumor shows extracapsular spread with positive margins unsuitable for reapproach, adjuvant CRT is recommended.^32^, ^33^, ^36^

Approximately 75%–81% of patients with OPSCC are diagnosed in stages III or IV,^40^, ^41^ in which the treatment strategy is challenging due to the greater extent of disease. For adequate exposure of the tumor for resection, different techniques are used based on the size and anatomic site of the neoplasm.^32^, ^33^ Macroscopic tumor-free margins of 1.5–2.0 cm are recommended in combination with frozen section analysis, which requires extensive surgery, but few centers have the expertise to perform it.^32^ Therefore, different cancer centers use surgery, RT, and CT in a variety of combinations for patients with advanced-stage OPSCC.^33^

Studies comparing the outcomes of surgical and medical treatment of patients with advanced-stage OPSCC have produced conflicting results.^27^, ^29^, ^30^ O’Connell et al.^27^ showed better 5-year survival rates with surgery followed by adjuvant combined CRT (71.1%) than with CRT (48.6%) or surgery followed by adjuvant RT (53.9%) for patients with advanced-stage disease. Díaz-Molina et al.^29^ compared oncologic and functional outcomes in patients with OPSCC treated with RT vs. surgery. In advanced stages, surgery was associated with a better prognosis than RT, with a 5-year disease- specific survival rates of 47% and 17%, respectively. Although the rate of successful return to oral food intake was higher in the surgical group, overall functional outcomes were similar in both groups.^29^ Kamran et al.^58^ found improved survival in primary surgery with RT ± CT for locally advanced OPC has an compared to primary radiation-based treatment, with a 3-year survival rates of 85.4% and 72.6%, respectively. In contrast, Soo et al.^30^ found no statistically significant difference in overall survival and 3-year disease-free survival rates when comparing surgery followed by adjuvant RT vs. CRT in patients with advanced-stage OPSCC, despite the significantly higher incidence of toxicity among patients receiving medical treatment. The heterogeneity of the results of studies comparing surgical vs. medical treatment may reflect a treatment selection bias in relation to the time when the study was performed. Until the 1990s, patients were predominantly treated with open surgery. CRT gained popularity in the 2000s, with TORS being introduced around 2010. TORS as a primary treatment for advanced oropharyngeal malignancy confers excellent survival and swallowing outcomes as Haughey et al.^59^ showed. The authors found 3-year overall survival, disease-specific survival, and disease-free survival were 86%, 88% and 82%, respectively. Local control was 97% and 87% of patients had normal swallowing or episodic dysphagia.

The present study showed no significant difference in disease-free survival between patients receiving medical and surgical treatment, regardless of OPSCC stage, which suggests similar complete remission rates with both approaches. However, patients who were not treated with surgery had shorter overall survival in both early and advanced stages of the disease. A possible explanation for this result may be the higher rate of deaths due to complications secondary to CT and RT compared with surgery. In this context, surgery would be the most suitable treatment for patients with OPSCC. However, the overall survival rate considers all-cause mortality, which includes deaths from cancer, treatment complications, or any other noncancer-related circumstances, and the FOSP data do not allow us to determine the proportion of deaths associated with the adverse effects of CT and RT. Therefore, we could not directly relate the lower overall survival of patients to treatment complications.

Our study has limitations inherent in a retrospective analysis. Comparison of nonrandomized data is associated with several problems including patient selection, incomplete medical records, and application of different staging systems. The FOSP has used different staging systems over the years: TNM fifth edition until 2005, TNM sixth edition from 2006 to 2013, and TNM seventh edition from 2014 onward. Therefore, patients with the same diagnosis could be classified into different stages depending on the year of diagnosis registration. Since the study used cases previous to the last TNM edition and does not differentiate positive or negative of HPV we cannot use this important prognostic factor trying to understand differences in treatment methods. Another limitation is the lack of information on comorbidities, type of surgery performed, and reason for medical treatment. The choice of nonsurgical treatment because of tumor unresectability or impaired functional status may determine selection bias, since patients within this profile are more likely to have adverse outcomes, regardless of treatment type. Additionally, we could not collect data on functional outcomes and quality of life, which are relevant factors in the comparison of treatment modalities.

The strengths of our study include a set of results that provide data on the population of patients with OPSCC in the state of São Paulo, with a sample representative of the demographic profile of the disease described in the literature. We identified a total of 8075 patients with OPSCC, with an increased OPSCC incidence in recent years and a higher prevalence in men (88.9%), in patients aged 50–70 years (66.0%), and of advanced-stage disease at diagnosis (86.3%). The mean time from diagnosis to initiation of treatment was 69.21 days, which might reflect the work overload of cancer centers in Brazil. It is important to note that 5.9% of patients died before treatment; and 1.5% did not start treatment due to advanced disease, lack of clinical conditions or other comorbities. Most patients received medical treatment (57.5%), which also may be a consequence of the delay in treatment initiation, with patients already presenting with unresectable tumors at the time of treatment decision- making. Our study also raises issues to be investigated in future research, such as the proportion of deaths related to the complications of each treatment modality.

Prospective randomized controlled trials that differentiate HPV-related and non-HPV-related tumors are warranted to provide consistent data on the best approach for patients with OPSCC.

## Conclusion

Within the limitations of the present study, our results provide epidemiological data on patients with OPSCC in the state of São Paulo, Brazil. There was no significant difference in disease-free survival between surgical and medical treatment, but patients who were not treated surgically had shorter overall survival. Prospective studies are warranted to assess whether these results are secondary to complications from the use of CT and RT.

## Authors’ contributions

Jonas Belchior Tamanini: Contributed to drafting the manuscript.

Fabio Portela Gazmenga: Contributed to drafting the manuscript.

Gustavo Mercuri: Contributed to revising the manuscript critically for important intellectual content.

Vanessa Carvalho de Oliveira: Contributed to revising the manuscript critically for important intellectual content.

Daniel Naves de Araújo: Contributed to drafting the manuscript; and critically revising it for important intellectual content.

Eduardo Vieira Couto: Contributed to drafting the manuscript; and critically revising it for important intellectual content.

Carlos Takahiro Chone: Contributed to the conception and design; and the acquisition, analysis, and interpretation of the data.

## Conflicts of interest

The authors declare no have conflicts of interest.
